# Sleep Disturbances Among Yazidi Survivors of the ISIS Genocide: Epidemiology, Neuropsychology, and Culturally Sensitive Interventions

**DOI:** 10.1002/brb3.71523

**Published:** 2026-06-16

**Authors:** Jan Ilhan Kizilhan, Zelal Ag, Qanta A. Ahmed, Alon Y. Avidan

**Affiliations:** ^1^ Institute For Transcultural Health Science Cooperative State University Stuttgart Germany; ^2^ Institute of Psychotherapy and Psychotraumatology University of Duhok Duhok Iraq; ^3^ Department of Medicine Associate Professor of Medicine (Clinical) NYU Grossman Long Island School of Medicine Mineola New York USA; ^4^ Department of Neurology and Sleep Medicine UCLA David Geffen School of Medicine Los Angeles California USA

**Keywords:** cultural psychiatry, insomnia, Narrative Exposure Therapy, nightmares, PTSD, sleep disturbances, Yazidi genocide

## Abstract

The Yazidi community endured an unprecedented genocide by the Islamic State (ISIS) in August 2014, resulting in mass killings, kinocide, abductions, ongoing disappearances, and sexual enslavement. The intense trauma led to high prevalence rates of posttraumatic stress disorder (PTSD), depression, anxiety, and profound sleep disturbances. This comprehensive article integrates epidemiological data, neurobiological mechanisms, and clinical case reports to elucidate sleep disorders among Yazidi survivors. We detail the historical context, transgenerational trauma, specific sleep‐related pathologies (insomnia, nightmares, parasomnias, and disrupted circadian rhythms), neurobiological underpinnings, cultural factors, and evidence‐based interventions (e.g., Narrative Exposure Therapy (NET); Cognitive Behavioral Therapy for Insomnia (CBT‐I)). Furthermore, we discuss the establishment and role of the Institute for Psychotherapy and Psychotraumatology (IPP) at the University of Duhok, outline barriers to care, and propose future research directions and policy recommendations.

## Graphical abstract

The figure presents an integrative model of sleep disturbances among Yazidi survivors of the 2014 genocide perpetrated by the Islamic State (ISIS). Genocide‐related trauma, including mass violence, sexual violence, displacement, and historical trauma, is associated with sustained neurobiological dysregulation, including hypothalamic–pituitary–adrenal (HPA)‐axis hyperarousal, rapid eye movement (REM) sleep fragmentation, and circadian rhythm disruption. These interacting neurobiological, cultural, and socio‐psychological mechanisms contribute to high rates of sleep disturbances, such as insomnia, nightmares, and parasomnias, which are closely linked to trauma‐related psychopathology and hinder psychological recovery. Culturally grounded, trauma‐informed, and sleep‐focused interventions, embedded in community engagement and policy‐level support, represent key pathways toward stabilization and long‐term healing in post‐genocide contexts.

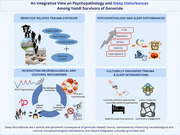

## 1 Introduction

The Yazidis are among the oldest religious communities of the Middle East, with roots that can be traced back to ancient Mesopotamian and Mithraic traditions (Kizilhan 2025). Their faith, transmitted orally over generations, shares historical links with Zoroastrianism while preserving a unique cosmology and rituals (Kreyenbroek 2009). Historically marginalized and persecuted as heretical, Yazidis have survived at least seventy‐four documented genocidal campaigns throughout history (Kizilhan 2016). The most recent, carried out by the so‐called Islamic State of Iraq and Syria (ISIS) in August 2014, constituted the most brutal assault on the community in recorded history, involving systematic killings, sexual enslavement, and mass displacement (Human Rights Council 2016).

On August 3, 2014, ISIS militants overtook Sinjar, murdering more than seven thousand Yazidi men, abducting approximately six thousand four hundred women and girls, and forcing thousands of families into surrounding mountains under dire conditions (Human Rights Council 2016; Tekin et al. 2016). These atrocities displaced over 360,000 Yazidis into camps across the Kurdistan Region, including Khanke, Chamishko, Qadya, Shariya, Bajidkandala, Bersive, Kabarto One, Kabarto Two, and Eissyan (Gerdau et al. 2017; WHO 2018). The combination of mass killings, sexual violence, and prolonged displacement resulted in exceptionally high rates of both acute and chronic post‐traumatic stress disorder (PTSD) among survivors (Kizilhan and Weigelt 2025, Tekin et al. 2016). Sleep disturbances—particularly chronic insomnia, recurrent nightmares, parasomnias, and circadian rhythm disruptions—emerged as core and often persistent symptoms within this trauma constellation.

At the time, the region lacked specialized mental health and sleep medicine infrastructure: no board‐certified sleep specialists or comprehensive sleep centers existed, and primary care physicians had minimal training in psychiatric or sleep‐related care (Hoffman and Weber 2020). As a result, Yazidi survivors received little to no systematic support for psychiatric conditions, let alone for the severe sleep disturbances that soon became widespread.

The aim of this article is to provide a state‐of‐the‐art, integrative overview of sleep disturbances among Yazidi survivors of the ISIS genocide, drawing on evidence from cross‐sectional surveys, qualitative interviews, clinical observations, and the development of culturally tailored interventions at the Institute for Psychotherapy and Psychotraumatology (IPP) at the University of Duhok. This article brings together perspectives from trauma psychology, sleep medicine, neurobiology, and cultural psychiatry to synthesize empirical findings with clinically grounded insights from post‐genocide and displacement contexts. It presents the historical and transgenerational background of the genocide, epidemiological patterns of sleep disorders, underlying neurobiological mechanisms, clinical case examples, and cultural factors and current interventions at the IPP before discussing future research directions and policy recommendations. By situating sleep disturbances within a broader biopsychosocial and cultural framework, this overview aims to inform integrated, context‐sensitive strategies for recovery and resilience. At the same time, this review provides a conceptual basis for future research on trauma‐related sleep disturbances in post‐genocide and kinocide contexts, with kinocide referring to the systematic targeting and destruction of family units as a distinct form of mass violence (Elkayam‐Levy et al. 2024).

## 2 Historical and Transgenerational Trauma

The psychological well‐being of Yazidi survivors of the ISIS genocide cannot be understood apart from the community's long history of persecution. Sleep disturbances among Yazidis reflect an intersection of historical trauma, cultural disruption, inherited memory, and neuropsychological vulnerability. These patterns are rooted in centuries of collective violence that have been preserved in communal identity and memory. Central to this historical consciousness is the concept of *Ferman*, referring to genocidal edicts and repeated extermination campaigns dating back to the Mongol, Safavid, and Ottoman periods (Kizilhan 2016; Kreyenbroek 2009). Oral traditions and religious ceremonies transmit narratives of forced displacement, property confiscation, and mass killings across generations, embedding persistent perceptions of existential threat, hypervigilance, and chronic anxiety (Gerdau et al. 2017; Kizilhan 2016).

Trauma may be transmitted intergenerationally through both biological and psychosocial mechanisms. Research on Holocaust survivors and their descendants demonstrates associations between extreme parental trauma and epigenetic modifications in offspring, including altered DNA methylation of glucocorticoid receptor–related genes, which may heighten hypothalamic–pituitary–adrenal (HPA) axis reactivity (Dashorst et al. 2019; Yehuda et al. 2016). These biological susceptibilities interact with disrupted attachment, parental hypervigilance, unresolved grief, and emotional numbing, shaping children's threat perception and affect regulation (Hinton and Kirmayer 2013; Lazar et al. 2006). Because sleep requires a basic sense of safety and the suspension of vigilance, persistent hyperarousal interferes with sleep initiation and maintenance. Over time, sustained arousal promotes avoidance of sleep and the sleep environment, contributing to insomnia, nightmares, and fragmented sleep, symptoms widely reported among Yazidi survivors.

These historically embedded vulnerabilities were violently reactivated during the ISIS atrocities of 2014, which represented a continuation and escalation of longstanding patterns of persecution. By labeling Yazidis as “infidels,” ISIS invoked jihadist fatwas to legitimize mass extermination (Kizilhan et al. 2020). Mass killings, sexual violence, and forced witnessing of atrocities reactivated collective memories of *Ferman*, reinforcing transgenerational fear and mistrust (Gerdau et al. 2017; Human Rights Council 2016; Kirmayer and Ryder 2016). Clinical reports and survivor narratives consistently describe severe trauma‐related nightmares and profound sleep disruption following these events, affecting both adults and children who directly witnessed violence or learned about it within their families (Ceri et al. 2016; Gerdau et al. 2017; Kurhan et al. 2024). Local psychologists and humanitarian workers were also exposed to repeated traumatic testimonies, placing them at risk for secondary traumatization (Daniels 2008).

Prolonged internal displacement further intensified these effects by disrupting social, cultural, and religious structures that traditionally supported resilience. Overcrowded camp conditions, resource scarcity, environmental exposure, and limited access to religious infrastructure interfered with collective rituals and social cohesion (Omarkhali 2016; WHO 2018). Gendered dimensions of trauma were particularly pronounced. Women who survived sexual violence frequently faced stigma as “dishonored,” leading to social isolation, shame‐related hypervigilance, feelings of mental contamination, and severe insomnia (Hassan et al. 2016). Clinical observations indicate that some survivors develop compulsive washing behaviors in response to contamination fears following sexual violation (Kizilhan 2010). Children exposed to cumulative and intergenerational trauma often displayed anxiety‐driven sleep disturbances, including fear of sleeping alone and recurrent nightmares of renewed persecution (Ceri et al., 2016).

## 3 Epidemiology of Sleep Disorders in the Aftermath of Genocide

Sleep disturbances are among the most consistently reported and functionally impairing consequences of exposure to war, mass violence, and prolonged traumatic stress. Across conflict‐affected populations, high rates of insomnia, nightmares, fragmented sleep, and circadian rhythm disruption have been documented and are closely linked to post‐traumatic stress disorder (PTSD) and related conditions (Jou and Pace‐Schott 2022). Studies from diverse war and displacement contexts, including Gaza and other conflict zones, indicate that civilians exposed to chronic insecurity and violence frequently experience persistent sleep disruption, particularly women and children (Hamamra et al. 2025; Al‐Khalil et al. 2024). Importantly, sleep disturbances in these settings are not only markers of psychological distress but also contribute to the maintenance and chronicity of trauma‐related psychopathology (Pandi‐Perumal et al. 2023).

Within this broader body of research, the Yazidi population represents a particularly severe and under‐researched case. Available epidemiological studies reveal a staggering psychological toll on Yazidi survivors. Among Yazidi children and adolescents assessed shortly after migration, disturbed sleep was the most prevalent psychiatric symptom, affecting more than 70% of participants (Ceri et al. 2016). In adult survivors of ISIS captivity, Grossman et al. (2019) reported a strong association between insomnia and complex post‐traumatic stress disorder (CPTSD), with over 95% of women meeting CPTSD criteria also fulfilling criteria for clinical insomnia. Depression and anxiety disorders were likewise highly prevalent, particularly among formerly enslaved women, driven by hypervigilance and fear of renewed violence (Mahmood et al. 2019; Tekin et al. 2016). Furthermore, recent surveys conducted in displacement settings and resettlement contexts indicate that rates of posttraumatic stress disorder (PTSD) remain particularly high. In a cross‐sectional study of 395 Yazidis—including non‐displaced individuals, internally displaced persons (IDPs), and individuals resettled in Germany—PTSD prevalence ranged from 43% to 57% among adult IDPs, while 92.7% of resettled women met PTSD criteria two years post‐resettlement (Kizilhan and Weigelt 2025).

Within this broader landscape of psychological distress, sleep disturbances emerged as among the most debilitating and persistent sequelae. Between 48% and 65% of adult Yazidi survivors reported insomnia, characterized by difficulties initiating and maintaining sleep (Ahmed et al. 2023; Kizilhan and Weigelt 2025). Recurrent nightmares were reported by more than half of adult participants (58%–72%), typically featuring vivid, distressing content related to mass executions or sexual violence (Kizilhan and Noll‐Hussong 2017). Among child survivors, parasomnias, such as confusional arousals or sleep‐related violent enactments, appeared in 12%–18% (Ahmed et al. 2023). Moreover, more than 70% of participants reported circadian rhythm disruptions—including delayed sleep phase syndrome—exacerbated by unstable daily routines, sustained insecurity during captivity, and adverse environmental conditions in displacement settings (Ahmed et al. 2023).

The epidemiological findings reviewed in this article are largely derived from cross‐sectional field studies conducted in conflict, displacement, and post‐displacement settings. Across the Yazidi‐specific literature, participants were predominantly recruited through community‐based access points, including refugee and IDP camps, host communities, clinical outreach programs, and resettlement initiatives. While these recruitment strategies are methodologically and ethically appropriate in highly vulnerable post‐genocide contexts, they necessarily limit the representativeness of samples with respect to the broader Yazidi population worldwide. Importantly, the convergence of findings across independent studies, age groups, displacement contexts, and assessment modalities indicates that severe and persistent sleep disturbances represent a robust and clinically relevant consequence of genocide‐related trauma among Yazidi survivors.

These findings were derived from self‐reports, standardized assessments, and objective measures, with instruments administered using established translation and clinician‐assisted oral interview procedures in Kurmanji, where appropriate. Validated instruments, including the Insomnia Severity Index and Pittsburgh Sleep Quality Index, assessed sleep symptoms, while the PCL‐5, PHQ‐9, HSCL‐25, and PIQ measured PTSD, depression, anxiety, and perceived injustice, respectively (American Psychiatric Association 2013; Kroenke et al. 2001; Perlis et al. 2005). In a subsample of 50 survivors, actigraphy provided objective confirmation of sleep pathology, revealing severe sleep fragmentation, reduced total sleep time (3–4 h/night), low sleep efficiency (<60%), and highly variable sleep–wake patterns (Ahmed et al. 2023). Qualitative interviews further contextualized these numbers. Camp psychologists and international NGOs corroborated these data, revealing survivors often ascribed insomnia to unquenchable fear, while attributing nightmares to spiritual or supernatural influences (Hassan et al. 2016).

Gender disparities add another layer to this picture. Female survivors reported disproportionately higher rates of insomnia (68% vs. 56% in males) and parasomnias (18% vs. 10% in males), likely reflecting gender‐specific traumas of rape and sexual enslavement (Tekin et al. 2016; Uysal et al. 2019). These disparities underscore the compounded burden faced by women and highlight the need for tailored interventions cognizant of compounded stigma and trauma. These findings also correlate with increased prevalence of sleep disorders in women compared to men at all stages of life, whether adulthood, pregnancy, perimenopause, or menopause in women.

## 4 Neurobiological Mechanisms Linking Trauma and Sleep Pathology

Sleep is a dynamic and metabolically highly active process. Sleep architecture is defined by sleep cycles oscillating from non‐rapid eye movement (NREM; stages N1–N3) into rapid eye movement (REM) sleep. Circadian rhythmicity of sleep is regulated by the brain's internal clock, the suprachiasmatic nucleus, which is entrained by the most potent zeitgeber‐time giver—daylight—but also greatly influenced by biological neurotransmitters and hormones, including melatonin and cortisol (Goel et al. 2013). While the function of sleep is not fully understood, it is a biological necessity for survival. Slow‐wave sleep (N3) facilitates restorative processes as well as the sense of rejuvenating sleep, while REM sleep integral to CNS development in the womb is clearly noted to consolidate emotional memory (Tononi and Cirelli 2014).

Beyond these functional roles, accumulating evidence indicates that sleep is also highly sensitive to stress‐related biological regulation. Experimental and clinical studies suggest associations between sleep loss, fragmentation, and epigenetic modifications, particularly DNA methylation changes in genes involved in circadian regulations, stress responses, and neural plasticity (Gaine et al. 2018; Gazerani 2020). While these epigenetic alterations do not change the DNA sequence, they do modulate gene expression and are discussed as potential mechanisms linking environmental stressors, including trauma and chronic sleep disruption, to neurobiological effects (Guo et al. 2025).

Following severe trauma, multiple neurobiological pathways contribute to sleep disruption. From an evolutionary standpoint, the severe sleep disruption seen after trauma, which results in profound insomnia, has been hypothesized to have a teleological benefit, seeking to avoid or reduce embedding of the traumatic memory by fragmenting and greatly reducing REM sleep. However, persistent disruption of REM and NREM sleep ultimately becomes maladaptive.

### 4.1 Hyperarousal and HPA‐Axis Overactivation

Traumatic stressors activate the hypothalamic–pituitary–adrenal (HPA) axis, elevating corticotropin‐releasing hormone and cortisol, which impair sleep onset. Fragmented sleep is further associated with escalated morning cortisol levels and a failure of cortisol dipping, delaying the normal drop in cortisol levels after healthy sleep. Chronic hyperarousal—a hallmark of PTSD—involves increased locus coeruleus–norepinephrine activity, promoting vigilance and reducing slow‐wave sleep, while heightened sympathetic tone elevates heart rate, diminishing restorative N3 sleep (Ross et al. 1994).

### 4.2 REM Sleep Dysregulation and Nightmares

PTSD is characterized by REM intrusion phenomena, including increased rapid eye movements and limb twitches indicative of dream enactment (Spoormaker and Montgomery 2008). The severe sleep fragmentation typical of PTSD also lends itself to REM sleep fragmentation and awakenings with intensely vivid, frightening, and disruptive dream recall, further driving the aversion to sleep, worsening insomnia, and magnifying the sleep disruption. Patients often awaken with severe hypervigilance. In clinical observations, particularly among survivors of war, individuals may request careful mechanisms of awakening to avoid a fight or flight response, which in military‐related PTSD can precipitate defensive responses. Dysregulation of cholinergic and monoaminergic neurotransmitters—where acetylcholine promotes REM, while serotonin and norepinephrine inhibit it—leads to more frequent, intense nightmares (Ross et al. 1994).

### 4.3 Circadian Rhythm Disruption

Elevated evening cortisol and nighttime hyperarousal, aversion to sleep, inhibit and delay dim light melatonin onset, resulting in the development of or exacerbation of delayed sleep phase syndrome (Arendt 2006). The absence of structured routines in camps and the mandated exposure to digital media radicalizing or psychologically torturing captives further delayed sleep, inhibited melatonin, and worsened the already desynchronized internal circadian clocks, impairing sleep consistency (Ahmed et al. 2023). These effects can be very persistent even beyond captivity.

### 4.4 Neuroimaging Correlates in Trauma‐Exposed Individuals

Structural imaging in PTSD often reveals reduced hippocampal volume—impairing memory consolidation during slow‐wave sleep—and enlarged amygdala, intensifying fear responses and REM intrusion (Bremner et al. 1997; Rauch et al. 2003). Functional neuroimaging demonstrates persistent amygdala and insula hyperactivation in response to trauma‐related cues, coupled with prefrontal cortex hypoactivity (Shin and Liberzon 2010). PET studies show decreased metabolic activity in medial prefrontal and anterior cingulate regions during sleep, correlating with fragmentation and nightmares (Germain et al. 2006).

## 5 Clinical Manifestations and Case Studies

Clinical presentations vary by age and context, reflecting the interplay of trauma severity, environmental stressors, and cultural beliefs. Two illustrative case studies highlight these dynamics.

### 5.1 Case 1: Adult Chronic Insomnia and Nightmare Disorder in an Adult Survivor

“Amina,” a 32‐year‐old former ISIS captive, witnessed the execution of her husband and children. Following her liberation, she experienced severe sleep onset and sleep maintenance insomnia (sleep latency > 90 min, multiple nocturnal awakenings) and nightly nightmares of being buried alive. Actigraphy over 2 weeks confirmed a short total sleep time of <4 h of sleep and reduced sleep efficiency, and an elevated fragmentation index (Ahmed et al. 2023). Neuropsychological testing showed memory and executive function deficits (Avidan and Heinzer 2020). Narrative Exposure Therapy (NET) and Imagery Rehearsal Therapy (IRT) helped rewrite traumatic memories and nightmares (Schauer et al. 2011; Krakow et al. 2001). Minimal pharmacotherapy (olanzapine and then prazosin) addressed hyperarousal and nightmares (Raskind et al. 2007). After four months, Amina reported improved sleep (5.5 h, 68% efficacy) and significant reduction in PTSD symptom severity (Kizilhan and Weigelt 2023).

### 5.2 Case 2: Pediatric Parasomnia and Sleep Anxiety

“Delal,” a 9‐year‐old boy drafted as an ISIS child soldier, witnessed the execution of his father. He suffered from parasomnias (e.g., violent movements and moaning demands to behead attackers) and refused to sleep alone, insisting on his mother's presence under her bed with binoculars, convinced ISIS militants still lurked. The child insisted on sleeping with a wooden sword, reportedly to feel safe from the constant fear that the terrorists might return, as a result of which he often stayed aware and developed a sleep rhythm disorder. He was noted to move the wooden sword purposefully in sleep suggesting dream enactment. Polysomnography revealed REM dysregulation (elevated REM percentage (28% which is often seen in depression), shortened REM latency (60 min)) as well as a hallmark of depression and a clear parasomnia episode marked by increased chin EMG and limb twitching (Ahmed et al. 2023). Treatment included child‐friendly NET with drawings and play (Neuner et al. 2004), dyadic parent–child therapy establishing a secure bedtime routine, and low‐dose melatonin (0.5 mg) to normalize circadian rhythms (Arendt 2006). Improved sleep hygiene with boundary setting on both cellphone usage and YouTube exposure were also imposed. Within three months, Delal began sleeping alone, parasomnias ceased, REM normalized to 22%, sleep anxiety resolved, and a total sleep time increased from 6.8 to 8.2 h and 88% to 91% sleep efficiency, paralleled by reduced PTSD symptoms and improved attention at school (Kizilhan and Weigelt 2025).

### 5.3 Environmental and Social Observations in Displacement Camps

Structural and social conditions in displacement camps, such as Shariya and Qadya, further undermine survivors’ sleep health and trauma symptoms. Persistent noise from generators and nocturnal disturbances impede restorative sleep (WHO 2018); artificial lighting fragments sleep cycles; and temperature extremes (summer heat > 40°C, winter cold < 10°C) exacerbate insomnia (Gerdau et al. 2017). Communal latrines and nighttime safety concerns trigger frequent awakenings. Furthermore, cultural norms contribute to gender‐specific behavior patterns. While men are discouraged from admitting fear or emotional distress, leading to underreporting of nightmares and reliance on self‐medication with alcohol or tranquilizers (Bride 2007), compulsive smoking, which is a typical stress response in the population, also worsens insomnia. Yazidi women, culturally governed in a shame and honor system, face continued stigma around sexual violence and rejection of any progeny conceived forcibly in their captivity, also deterring formal mental health care (Hassan et al. 2016). These factors create a context in which sleep disturbances thrive.

## 6 Cultural and Sociopsychological Factors in Sleep Pathology

Yazidi cultural beliefs and social dynamics profoundly shape how sleep disturbances are experienced and addressed. In this context sleep is not merely a biological function but also entails spiritual and communal significance. Nightmares are often interpreted as intrusions by jinn (evil spirit) (Omarkhali 2016) and may be seen as evidence of “weak faith” by survivors (Kirmayer and Ryder 2016) or spiritual weakness by families (Schouler‐Ocak 2015). Such interpretations reinforce fear of sleep and discourage discussing symptoms, encouraging survivors to endure suffering in silence, particularly in displacement settings where nightly rituals such as communal prayers and religious melodies that once served to ward off nightmares were disrupted by displacement (Hassan et al. 2016).

The collectivist orientation of Yazidi society traditionally provides communal support through extended families and religious rituals (Kreyenbroek, 1995). However, displacement has fractured these networks, as family members now inhabit different camps or countries, weakening established resilience mechanisms (Gerdau et al. 2017). Within this vacuum, gender roles and stigma present further barriers: men, expected to remain stoic, underreport nightmares and may self‐medicate with alcohol (Bride 2007), while women, who endured sexual violence, feel “dishonored,” causing shame and isolation, which exacerbates hypervigilance and insomnia (Lončar et al. 2006; Tekin et al. 2016). Recent findings showed that feelings of humiliation and shame among Yazidi women who survived sexual violence are strongly associated with dissociative symptoms such as dissociative seizures, which in turn correlate with severe sleep problems, including insomnia and nightmares (Kizilhan et al. 2020). These dynamics underline how shame not only contributes to social withdrawal but also directly intensifies sleep pathology through mechanisms of dissociation and hyperarousal. Hence, despite greater openness to share distress with neighbors or religious leaders, women seldom access professional care due to stigma but also childcare duties and household obligations (Hassan et al. 2016; Kizilhan et al. 2020).

Language barriers complicate diagnosis and treatment. No direct Kurmanji terms for “insomnia” or “nightmare disorder” exist; instead, idioms like “heart not at rest at night” must suffice (Kirmayer and Ryder 2016). Translating instruments without extensive oral explanation, such as the Insomnia Severity Index, can lead to misinterpretation (Perlis et al. 2005). Hence, clinicians rely on bilingual therapists to contextualize questions (Kizilhan and Weigelt 2023). Moreover, somatization is common: survivors often report headaches, chest pain, or gastrointestinal distress rather than acknowledging sleep‐related or psychological symptoms (Kirmayer and Ryder 2016). Clinicians must remain vigilant, recognizing that fragmented, non‐linear narratives reflect cultural storytelling styles, not deception (Droidek 2010).

These cultural and sociolinguistic dynamics not only shape how sleep disturbances are expressed but also determine whether, how, and from whom help is sought. Conventional, Western‐oriented approaches to sleep pathology often fail to reach Yazidi survivors, as the absence of culturally resonant terminology, reliance on idioms of distress, and pervasive stigma around trauma and gender‐based violence create major obstacles to effective diagnosis and treatment. At the same time, displacement has eroded many of the community's own resilience mechanisms, leaving survivors caught between disrupted traditions and inaccessible biomedical care. Effective interventions must therefore be linguistically accessible, culturally grounded, and sensitive to the community's spiritual, social, and gender‐specific realities. One notable effort to embody this approach is the Institute for Psychotherapy and Psychotraumatology (IPP), established in 2017 at the University of Duhok.

## 7 Institute for Psychotherapy and Psychotraumatology Interventions

The Institute for Psychotherapy and Psychotraumatology (IPP), founded in 2017 at the University of Duhok through collaboration with the Baden‐Württemberg Ministry of Science, Research, and Arts (Adorjan et al. 2017), represents a rare and regionally grounded model for trauma‐informed care in post‐genocide settings. It combines clinical training, culturally adapted interventions, and outreach services to address the psychological aftermath of the ISIS atrocities, with a growing emphasis on sleep disturbances as a core symptom domain.

### 7.1 Training and Outreach

The 3‐year Master of Advanced Studies in Psychotherapy and Psychotraumatology (MASPP), offered in English and Kurdish, equips local psychologists with theoretical and practical skills in trauma treatment, including cognitive behavioral therapy, exposure therapy, Narrative Exposure Therapy (NET; Schauer et al. 2011), basic sleep medicine, and cultural psychiatry (Beckmann et al. 2022). Supervised internships in displacement camps allow students to apply these methods in real‐world conditions (Kizilhan and Weigelt 2023). In parallel, mobile teams deliver psychological first aid and sleep hygiene education to remote villages and camps in collaboration with local NGOs and United Nations agencies (WHO 2018). A training‐of‐trainer approach expands local capacity, while tele‐supervision connects Kurdish‐speaking clinicians with international specialists in sleep medicine and psychotraumatologists in Germany and the United States for ongoing case consultation (Adorjan et al. 2017, Kornwachs et al. 2024). MASPP students also conduct sleep‐related research projects, documenting outcomes of culturally adapted interventions (Beckmann et al. 2022).

### 7.2 Clinical Services

IPP's clinical outreach encompasses an outpatient clinic offering PTSD assessments using combined standardized trauma assessments (PHQ‐9; Kroenke et al. 2001; PCL‐5; Weathers et al. 2013; HSCL‐25; Derogatis and Lipman, 1977), sleep disorder screening (locally adapted Pittsburgh Sleep Quality Index; Buysse et al. 1989), and individual and group therapy. Pediatric screening employs the Children's Sleep Habits Questionnaire (Owens et al. 2000), administered with Kurmanji translation assistance. Survivors with significant insomnia or poor sleep quality are referred for further evaluation using portable actigraphy devices, as no in‐lab polysomnography is available (Ahmed et al. 2023; Avidan and Heinzer 2020).

### 7.3 Therapeutic Adaptations

What distinguishes IPP's approach is the integration of evidence‐based therapies with culturally specific adaptations. These psychotherapeutic modalities at IPP include NET, guiding survivors to construct coherent life narratives integrating traumatic events, thereby reducing fragmentation and nightmares (Kornwachs et al. 2024). Therapists incorporate Yazidi religious metaphors and rituals to affirm experiences, and group NET sessions foster communal support (Beckmann et al. 2022). CBT‐I is delivered with pictograms for varying literacy levels and involves family members for stimulus control and sleep restriction strategies (Perlis et al. 2005). IRT helps survivors rewrite nightmares using culturally relevant imagery—such as the sacred peacock angel or Yazidi temples—to replace traumatic content with symbols of safety (Krakow et al. 2001).

### 7.4 Pharmacological and Psychoeducational Support

While pharmacotherapy adheres to international PTSD guidelines, adaptations reflect local resource constraints. First‐line medication for PTSD‐related nightmares is low‐dose prazosin (Raskind et al. 2007); trazodone is used for insomnia co‐occurring with depression (Zhao et al. 2025), and melatonin (2–5 mg) helps normalize circadian rhythms when survivors lack structured routines (Arendt 2006). Benzodiazepines are generally avoided due to dependency risk and potential PTSD symptom exacerbation (Zhao et al. 2025). Complementing pharmacological care, monthly group psychoeducation workshops are organized to address sleep physiology, hygiene practices, and stress reduction (Perlis et al. 2005), supported by printed and visual leaflets in Kurmanji. Furthermore, Yazidi religious leaders are engaged to endorse sleep hygiene as a form of spiritual self‐care, improving community acceptance (Hassan et al. 2016).

### 7.5 Barriers and Challenges

Despite these efforts, significant structural barriers remain. Only ten actigraphy units serve the entire Kurdistan Region, and no in‐lab polysomnography exists, requiring reliance on subjective reports and portable devices (Ahmed et al. 2023). Stockouts of prazosin and trazodone leave survivors without consistent access to pharmacotherapy. The psychologist‐to‐population ratio remains approximately 1:10,000 in camps (Kurdistan Region Statistics Office 2023). Stigma equating sleep complaints with spiritual weakness discourages help‐seeking, and labeling individuals as “mad” prompts concealment (Schouler‐Ocak 2015). Security concerns—sporadic violence, curfews, and poor infrastructure—disrupt therapy schedules and limit mobile team visits, particularly during winter when roads become impassable (Kornwachs et al. 2024; WHO 2018).

Nonetheless, IPP illustrates both the potential and the limits of trauma‐informed, culturally sensitive sleep interventions in conflict‐affected regions. Its experience offers key lessons for replication while also highlighting the urgent need for systemic investment in sleep and mental health infrastructure.

## 8 Research Directions and Gaps

Despite promising interventions, significant research gaps continue to limit the development of targeted, effective care. Addressing these gaps is critical to improving sleep‐related outcomes and ensuring sustainable, culturally sensitive treatment models.

From a neurobiological perspective, longitudinal studies are required to examine the stability, reversibility, and clinical relevance of trauma‐related HPA‐axis alterations and epigenetic modifications, particularly among Yazidi children born after the genocide (Yehuda et al. 2016). Emerging data suggests that sleep disruption and prolonged stress may be associated with DNA methylation changes and altered stress regulation; however, the persistence, developmental timing, and functional significance of these alterations remain poorly understood (Gaine et al. 2018; Guo et al. 2025). Genetic analyses focusing on polymorphisms such as FKBP5 and BDNF may identify biomarkers of vulnerability or resilience (McGowan et al. 2009). Addressing these gaps is essential to determine whether neurobiological and epigenetic markers can inform risk stratification, prevention, or recovery trajectories in trauma‐affected populations. In addition, functional neuroimaging in displacement settings could elucidate trauma‐related brain activation patterns during sleep and wake (Germain et al. 2008).

Clinically, randomized controlled trials comparing therapeutic modalities remain scarce. Direct comparison between CBT‐I and prazosin for PTSD‐related sleep disturbances (Raskind et al. 2007), as well as multicenter trials assessing combined NET and IRT versus standard CBT (Krakow et al. 2001; Schauer et al. 2011), would inform evidence‐based treatment pathways. Feasibility studies launching mobile sleep medicine teleclinics in remote camps would inform scalable interventions (Avidan and Heinzer 2020).

Sociocultural research must also deepen its focus. Evolving dream interpretations among Yazidis in camps versus diaspora remain understudied (Omarkhali 2016), as does the impact of how returning to Sinjar affects sleep health. Gender‐specific studies should address sleep interventions tailored to female survivors of sexual violence (Tekin et al. 2016; Uysal et al. 2019).

Finally, policy and implementation science efforts must evaluate the cost‐effectiveness of establishing regional sleep medicine centers in Erbil and integrate sleep screening into primary care for IDPs (Weigelt and Kizilhan 2025). Educational campaigns to destigmatize mental health and sleep disorders warrant systematic evaluation (Hassan et al. 2016).

## 9 Policy Recommendations and Integrated Care Models

Strengthening regional sleep infrastructure is paramount to ensure long‐term and systemic investment in culturally anchored, trauma‐informed sleep care. Establishing one central sleep disorders center in Erbil, linked via telehealth to satellite clinics in Duhok and Sulaymaniyah, would provide centralized expertise and training (Hoffman and Weber 2020). Training at least ten local sleep technicians within 2 years and procuring 20 portable polysomnography units, ideally in the form of the Zoll Itamar WatchPat ONE technology, which can be recycled and also remotely dispensed, would enhance diagnostic capacity (Kurdistan Region Statistics Office 2023). Developing sleep disorder education modules in the Kurdish language for community health workers would promote early identification and referral (Kizilhan and Weigelt 2023).

Equally important is the integration of mental health and sleep services. Embedding validated sleep screening instruments—such as the Insomnia Severity Index and Pittsburgh Sleep Quality Index—into routine PTSD assessments at IPP clinics, administered through clinician‐assisted oral interviews with cultural contextualization, ensures that sleep disorders are not overlooked (Perlis et al. 2005; Buysse et al. 1989). A collaborative care model, in which psychiatrists, psychologists, sleep specialists, and primary care physicians work together, would optimize treatment outcomes (Kizilhan and Weigelt 2023). In camp schools, weekly sleep education sessions teaching sleep hygiene and relaxation techniques could foster healthier sleep habits among children (Owens et al. 2000).

For these interventions to succeed, they must be grounded in cultural sensitivity and community engagement as core principles. Recruiting and training 50 “sleep ambassadors” from Yazidi internally displaced populations—survivors with lived experience—will facilitate dissemination of sleep hygiene practices in a culturally congruent manner (Weigelt and Kizilhan 2025). Engaging religious leaders to frame sleep care as a sacred duty aligns interventions with local values (Hassan et al. 2016). Radio programs in Kurmanji featuring survivor testimonies about sleep recovery can reduce stigma and encourage care‐seeking (Kizilhan and Weigelt 2023).

Finally, legal advocacy must secure sustained funding from the Kurdistan Regional Government and international donors for integrated mental health–sleep programs (WHO 2018). Expanding coverage of mental health and sleep care under the Yazidi Survivors Law (Republic of Iraq 2021) will reduce financial barriers. Supporting international tribunals to prosecute ISIS crimes serves justice and strengthens restorative processes that promote community healing and individual psychological recovery (Nadler and Shnabel 2015).

Taken together, these policy measures represent a roadmap for embedding culturally sensitive sleep care into post‐genocide recovery, combining infrastructure, integration, community empowerment, and justice.

## 10 Discussion and Summary

The 2014 ISIS genocide inflicted a catastrophic psychological toll on the Yazidi community, with sleep disturbances emerging as a central barrier to recovery. Across conflict‐affected populations, and particularly among Yazidi survivors, chronic insomnia, frequent nightmares, parasomnias, and circadian disruptions are not only prevalent symptoms but also mechanisms that can maintain and amplify trauma‐related psychopathology (Ahmed et al. 2023; Ceri et al. 2016; Germain et al. 2008; Kizilhan and Weigelt 2025). Building on the epidemiological evidence and underlying mechanisms reviewed above, this paper advances a unified conceptual framework in which sleep represents a key intersection of neurobiological dysregulation, historical trauma, and sociocultural context. From a neurobiological perspective, trauma‐related sleep pathology reflects sustained dysregulation across interacting systems, including HPA‐axis overactivation, alterations in REM and slow‐wave sleep, circadian misalignment, and alterations across limbic and prefrontal systems that compromise emotional memory processing and stress regulation (Germain et al. 2006; Ross et al. 1994). Yet these biological changes do not unfold in isolation. In displacement settings, ongoing environmental stressors interact with biological vulnerability, exacerbating hyperarousal and circadian disruption and thereby prolonging sleep pathology beyond the initial traumatic exposure (Gerdau et al. 2017, Kurhan et al. 2024). These observations support an integrative view in which biological and contextual factors operate dynamically rather than as separate explanatory layers.

Within the Yazidi context, sleep disturbances are also embedded in historical trauma, collective memory, and culturally shaped interpretations of distress. Nightmares, hypervigilance, and insomnia are frequently understood through spiritual or moral frameworks rather than biomedical models, which can influence help‐seeking behavior, treatment engagement, and perceived meanings of recovery (Hassan et al. 2016; Kirmayer and Ryder 2016; Omarkhali 2016; Schouler‐Ocak 2015). Therefore, models of trauma‐related sleep pathology must be interpreted within their cultural context, as systems of meaning and social conditions shape symptom expression, coping strategies, and recovery trajectories (Kirmayer and Ryder 2016; Kizilhan and Weigelt 2023; Perlis et al. 2005).

In response to these complex challenges, the experience of the IPP in Duhok illustrates how sleep‐focused care can be implemented into culturally tailored, trauma‐informed services, specifically under resource constraints. Interventions such as NET and IRT, combined with CBT‐I adapted for low‐literacy contexts, represent feasible approaches to address both sleep disturbances and broader trauma‐related symptoms (Krakow et al. 2001; Perlis et al. 2005; Schauer et al. 2011). In addition, mobile outreach and tele‐supervision models extend care to remote camps and facilitate ongoing capacity‐building where specialist resources are limited (Adorjan et al. 2017; Avidan and Heinzer 2020). Still, resource constraints, stigma, and security challenges hinder widespread implementation. Research gaps remain, particularly regarding longitudinal neurobiological trajectories, culturally specific sleep beliefs, and gender‐sensitive treatment modalities (Kirmayer and Ryder 2016; Yehuda et al. 2016).

To achieve sustainable recovery, integrated care models are essential and must bridge sleep medicine and mental health services, embed culturally sensitive approaches, and secure stable funding (Kizilhan and Weigelt 2023). This requires coordinated policy initiatives that should prioritize establishing sleep centers, training local providers, and integrating sleep screening into routine PTSD care (Hoffman and Weber 2020; Kurdistan Region Statistics Office 2023). Equally important is community engagement—through “sleep ambassadors,” religious leader involvement, and radio outreach—that can reduce stigma and promote help‐seeking (Weigelt and Kizilhan 2023; Hassan et al. 2016). Concurrently, robust transitional justice efforts must validate survivors’ experiences, promoting healing through acknowledgment and accountability (Nadler and Shnabel 2015; Human Rights Council 2016).

While the Yazidi genocide may appear isolated, atrocities and mass violence continue elsewhere in the world. Comparable patterns of trauma‐related sleep disturbances have been reported among civilians exposed to such violence, with recent evidence from Israel showing marked increases in insomnia, short sleep, and sleep medication use during the 2023–2024 Israel–Hamas war (Zak et al. 2025). This underscores the global relevance of sleep‐focused trauma care and highlights opportunities for international collaboration, particularly given Israel's well‐established expertise in sleep medicine.

Overall, sleep health is inextricably linked to psychological recovery among Yazidi genocide survivors. Addressing the multifaceted interplay of historical trauma, neurobiology, and cultural context through integrated, culturally grounded, and justice‐oriented strategies is essential. Such approaches will not only alleviate sleep disturbances but also strengthen the Yazidi community's capacity for resilient, long‐term healing.

## Author Contributions

**Jan Ilhan Kizilhan**: conceptualization, investigation, methodology, resources, supervision, writing – original draft, writing – review and editing. **Zelal Ag**: investigation, writing – review and editing. **Qanta A. Ahmed**: investigation, resources, writing – review and editing. **Alon Y. Avidan**: Investigation, Resources, writing – review and editing

## Funding

The authors have nothing to report.

## Conflicts of Interest

The authors declare no conflicts of interest.

## Data Availability

Data sharing not applicable to this article as no datasets were generated or analyzed during the current study
